# Supplementary material for: Study protocol for COvid-19 Vascular sERvice (COVER) study: The impact of the COVID-19 pandemic on the provision, practice and outcomes of vascular surgery
Source: PLoS One. 2020 Dec 30;15(12):e0243299. doi: 10.1371/journal.pone.0243299 (PMC7773264; doi:10.1371/journal.pone.0243299)
Supplement: S1 File — (DOCX) [file pone.0243299.s001.docx]

| **Stroke** | | |
| --- | --- | --- |
| 30 days | 6 months | 12 months |
| Carotid endarterectomy completed | Carotid endarterectomy completed | Carotid endarterectomy completed |
| Carotid stenting completed | Carotid stenting completed | Carotid stenting completed |
| TIA |  | TIA |
| Amourosis Fugax | Amourosis Fugax | Amourosis Fugax |
| Re-admisssion | Re-admisssion | Re-admisssion |
| disabling stroke | disabling stroke | disabling stroke |
| non-disabling Stroke | non-disabling Stroke | non-disabling Stroke |
| Death |  | Death |
| Wound infection not requiring admission | Wound infection not requiring admission | Wound infection not requiring admission |
| Wound infection requiring admission | Wound infection requiring admission | Wound infection requiring admission |
| Myocaridal infarction | Myocaridal infarction | Myocaridal infarction |
| COVID 19 pneumonia | COVID 19 pneumonia | COVID 19 pneumonia |
| Other respiratory complication | Other respiratory complication | Other respiratory complication |
| COVID positive | COVID positive | COVID positive |
| Venous thrombosis / PE / DVT event | Venous thrombosis / PE / DVT event | Venous thrombosis / PE / DVT event |

S1 File.

Preliminary list of condition specific outcomes to be reported at 30 day, 6 and 12 month follow-up

| **CLTI** | | |
| --- | --- | --- |
| **30days** | **6 months** | **12months** |
| revascularisation Intervention now scheduled within 2 weeks or completed | revascularisation Intervention now scheduled within 2 weeks or completed | revascularisation Intervention now scheduled within 2 weeks or completed |
| had major amputation | had major amputation | had major amputation |
| Re-admisssion | Re-admisssion | Re-admisssion |
| Stroke | Stroke | Stroke |
| Myocaridal infarction | Myocaridal infarction | Myocaridal infarction |
| Death | Death | Death |
| Wound infection not requiring admission | Wound infection not requiring admission | Wound infection not requiring admission |
| Wound infection requiring admission | Wound infection requiring admission | Wound infection requiring admission |
| COVID 19 pneumonia | COVID 19 pneumonia | COVID 19 pneumonia |
| Other respiratory complication | Other respiratory complication | Other respiratory complication |
| Graft or stent Occlusion / thrombosis / patch thrombosis | Graft or stent Occlusion / thrombosis / patch thrombosis | Graft or stent Occlusion / thrombosis / patch thrombosis |
| Limb-loss | Limb-loss | Limb-loss |
| COVID positive | COVID positive | COVID positive |
| Venous thrombosis / PE / DVT event | Had revision amputation | Had revision amputation |

| **Abdominal aortic aneurysm** | | |
| --- | --- | --- |
| **30days** | **6 months** | **12months** |
| Intervention now scheduled within 2 weeks / or compelted | Intervention now scheduled within 2 weeks / or compelted | Intervention now scheduled within 2 weeks / or compelted |
| Had repair reached threshold services resumed | Had repair reached threshold services resumed | Had repair reached threshold services resumed |
| Had repair due to symptoms/rapidgrowth | Had repair due to symptoms/rapidgrowth | Had repair due to symptoms/rapidgrowth |
| Re-admisssion | Re-admisssion | Re-admisssion |
| Stroke | Stroke | Stroke |
| Myocaridal infarction | Myocaridal infarction | Myocaridal infarction |
| Death | Death | Death |
| Wound infection not requiring admission | Wound infection not requiring admission | Wound infection not requiring admission |
| Wound infection requiring admission | Wound infection requiring admission | Wound infection requiring admission |
| COVID 19 pneumonia | COVID 19 pneumonia | COVID 19 pneumonia |
| Other respiratory complication | Other respiratory complication | Other respiratory complication |
| Graft Occlusion (open or endograft) | Graft Occlusion (open or endograft) | Graft Occlusion (open or endograft) |
| EVAR/Endograft thrombosis / Target vessel occlusion | EVAR/Endograft thrombosis / Target vessel occlusion | EVAR/Endograft thrombosis / Target vessel occlusion |
| Major lower limb amputation | Major lower limb amputation | Major lower limb amputation |
| Bowel ischaemia / Bowel surgery | Bowel ischaemia / Bowel surgery | Bowel ischaemia / Bowel surgery |
| COVID positive | COVID positive | COVID positive |
| Venous thrombosis / PE / DVT event | Venous thrombosis / PE / DVT event | Venous thrombosis / PE / DVT event |

| **Diabetic Foot Infection** | | |
| --- | --- | --- |
| **30days** | **6 months** | **12months** |
| Due or compelted angioplasty / revascularisation | Due or compelted angioplasty / revascularisation | Due or compelted angioplasty / revascularisation |
| Return to theatre / salvage procedure | Return to theatre / salvage procedure | Return to theatre / salvage procedure |
| Endovascular re-operation / salvage | Endovascular re-operation / salvage | Endovascular re-operation / salvage |
| Return to level 2/3 | Return to level 2/3 | Return to level 2/3 |
| had major amputation | had major amputation | had major amputation |
| had minor amputation | had minor amputation | had minor amputation |
| Re-admisssion | Re-admisssion | Re-admisssion |
| Stroke | Stroke | Stroke |
| Myocaridal infarction | Myocaridal infarction | Myocaridal infarction |
| Death | Death | Death |
| Wound infection/complication not requiring admission | Wound infection/complication not requiring admission | Wound infection/complication not requiring admission |
| Wound infection requiring admission | Wound infection requiring admission | Wound infection requiring admission |
| COVID 19 pneumonia | COVID 19 pneumonia | COVID 19 pneumonia |
| Other respiratory complication | Other respiratory complication | Other respiratory complication |
| Graft or stent Occlusion / thrombosis / patch thrombosis | Graft or stent Occlusion / thrombosis / patch thrombosis | Graft or stent Occlusion / thrombosis / patch thrombosis |
| COVID positive | COVID positive | COVID positive |
| Venous thrombosis / PE / DVT event | Venous thrombosis / PE / DVT event | Venous thrombosis / PE / DVT event |
