# Supplementary material for: Study protocol for COvid-19 Vascular sERvice (COVER) study: The impact of the COVID-19 pandemic on the provision, practice and outcomes of vascular surgery
Source: PLoS One. 2020 Dec 30;15(12):e0243299. doi: 10.1371/journal.pone.0243299 (PMC7773264; doi:10.1371/journal.pone.0243299)
Supplement: S2 File — (DOCX) [file pone.0243299.s002.docx]

# S2 File

**International/continental comparison analysis**

We will perform international/continental comparisons, to describe relative change in practice from normal. This will be achieved by allocating a score of 0/1/2/3 to each possible answer for each service evaluation question. A score will be allocated based on the perceived relative service reduction (with ‘0’ representing no change and ‘3’ representing the most significant change). For example, for the question: “have you changed your operative practice for elective AAA survey?” the answer ‘no change to practice’ will automatically scored 0, whereas the answer ‘limiting surgery to >7cm asymptomatic AAA’, a significant change, could be scored 1/2/3. A score for each survey question answer will be independently provided by 12 COVER team members (all vascular specialists). The mean value from these responses will then be used to quantify the overall change in vascular service provision for each responding unit.
