# Supplementary material for: Study protocol for COvid-19 Vascular sERvice (COVER) study: The impact of the COVID-19 pandemic on the provision, practice and outcomes of vascular surgery
Source: PLoS One. 2020 Dec 30;15(12):e0243299. doi: 10.1371/journal.pone.0243299 (PMC7773264; doi:10.1371/journal.pone.0243299)
Supplement: Attachment — Submitted filename: Reviewer comment_PONE-D-20-13638_COVER.docx [file pone.0243299.s003.docx]

**Comment to the Authors**

Title: Study protocol for COvid-19 Vascular sERvice(COVER) study: The impact of the COVID-19 pandemic on the provision, practice, and outcomes of vascular surgery

**General comment**

 It is an original and timely study protocol. The proposed methodology is scientifically and technically sound. Minor revision with emphasis on methodological re-structuring is required. I hope the detail review comments and suggestions will help you to improve your protocol and to consider some unforeseen challenges during the implementation of your study proposal.

**Specific comments**

**Abstract –** Well summarized

**Funding statement (L32) -**  It seems VERN is part of the Funding organization(VSGBI).

If so, there may be a question of competing interest. You may need to clarify the difference between VERN and Vascular Society of Great Britain.

**Method –** Need to restructure the method section – I suggest having the ‘objective’ section before the method so that you can put the general and specific objectives. The objectives are fragmented and there are lots of redundancies.

**L130 – 138**, Move the paragraph about the “overview of VERN” into the introduction part. Probably at the end of the introduction and before method.

**L139-141** – Move to the objective section or just put it at the end of the introduction.

Strat method with L141 “ This study has 3 tiers ….”

**L152** – Once you put the general and specific objectives for each tier above, you may not have to repeat the objectives for each tier. If you prefer to put under each tier, just avoid repetitions.

**L152** - I think if there is no secondary objectives or secondary outcomes, no need to level as ”Primary”. Apply this for the following tier as well.

**L166 -179** – Too many outcomes and some of these are activities that may define the listed outcomes. For example, conducting multi-disciplinary team meetings is an activity.

**L185-189** – See comments above about objectives for each tier.

**L193-197,** Patient enrolment

- How about the exclusion criteria? Do you have any?
- Will you include unknown status for COVID-19? Refusals for testing?
- You are to include prospective patients.
- How do you minimize providers' bias?
- Will you let the providers/Vascular specialists know about the study?

**L216-224** – You may need to have an “Operational definition” section to define the outcome variables. For example,

- How do you measure morbidity? Morbidity related to Vascular disease, related to intervention, COVID-19, or co-morbidities?
- How many days of sickness is considered as morbidity? Measure subjectively or objectively?
- The same thing does for Re-admission.
- Within how many days after discharge is considered as re-admission?
- With the same case or re-admission for any case?

**L234** – You will invite vascular specialists only to complete the survey. However, the burden of COVID-19 also affects other professionals like nurses, anesthesiologists, and surgical technologists. Why do you propose to include only vascular specialists in this study?

**Sampling and sample size determination** - Although it is stated under limitation, it is better to explain how to include participants for both patients and vascular specialists. You can also put a sample size but justify it.

- Do you have any inclusion criteria for the Vascular Specialists?
- How do you control/consider cofactors?
- Do you think all the situations, except related to COVID-19, are comparable pre- and during the pandemics?
- Please put some assumptions that will justify your comparative analysis before and during the COVID-19 pandemics.

**References**

- Some references missed the publication date or accessed date. Reference 1&12.
- I can't see the in-text citation for references 12&13. I am not sure but just check all the in-text citations appear in the reference lists and vice-versa.
