# Supplementary material for: Study protocol for COvid-19 Vascular sERvice (COVER) study: The impact of the COVID-19 pandemic on the provision, practice and outcomes of vascular surgery
Source: PLoS One. 2020 Dec 30;15(12):e0243299. doi: 10.1371/journal.pone.0243299 (PMC7773264; doi:10.1371/journal.pone.0243299)
Supplement: Attachment — Submitted filename: Response_to_reviewers.docx [file pone.0243299.s004.docx]

1. Please ensure that your manuscript meets PLOS ONE's style requirements, including those for file naming. The PLOS ONE style templates can be found at

<https://journals.plos.org/plosone/s/file?id=wjVg/PLOSOne_formatting_sample_main_body.pdf> and

<https://journals.plos.org/plosone/s/file?id=ba62/PLOSOne_formatting_sample_title_authors_affiliations.pdf>

2. We note that you have stated that you will provide repository information for your data at acceptance. Should your manuscript be accepted for publication, we will hold it until you provide the relevant accession numbers or DOIs necessary to access your data. If you wish to make changes to your Data Availability statement, please describe these changes in your cover letter and we will update your Data Availability statement to reflect the information you provide.

**This has been changed – there is no data being generated directly from this paper. Any subsequent results from the study, if accepted by Plos One for publication, will be made fully accessible.**

3. One of the noted authors is a group or consortium [Vascular and Endovascular Research
Network (VERN) Executive Committee]. In addition to naming the author group, please list the individual authors and affiliations within this group in the acknowledgments section of your manuscript. Please also indicate clearly a lead author for this group along with a contact email address.

**This has been changed and formatted as per your style requirements.**

4. Please include captions for your Supporting Information files at the end of your manuscript, and update any in-text citations to match accordingly. Please see our Supporting Information guidelines for more information: http://journals.plos.org/plosone/s/supporting-information.

**Supporting material has been submitted with this application and referenced in the text.**

5. Your ethics statement should only appear in the Methods section of your manuscript. If your ethics statement is written in any section besides the Methods, please delete it from any other section.

**This has been deleted from all sections other than the methods.**

# Responses to reviewers

**Comment to the Authors**

Title: Study protocol for COvid-19 Vascular sERvice(COVER) study: The impact of the COVID-19 pandemic on the provision, practice, and outcomes of vascular surgery

**General comment**

 It is an original and timely study protocol. The proposed methodology is scientifically and technically sound. Minor revision with emphasis on methodological re-structuring is required. I hope the detail review comments and suggestions will help you to improve your protocol and to consider some unforeseen challenges during the implementation of your study proposal.

***Thank you for your comments, we appreciate the time taken to read our protocol. We have responded to them and that the manuscript has been updated to reflect your suggestions.***

**Specific comments**

**Abstract –** Well summarized

**Funding statement (L32) -**  It seems VERN is part of the Funding organization(VSGBI).

If so, there may be a question of competing interest. You may need to clarify the difference between VERN and Vascular Society of Great Britain.

**Mention of supporting organisations have been completely moved to the acknowledgements. We have included the following statements to clearly document the separation between the two entities:**

1. **Line 53, abstract: ‘an independent, international vascular research collaborative’**
2. **Line 119: ‘established independent, international’**
3. **In the funding statement: ‘The funders have had no role in the design, implementation, analysis or write up of the study.’**

**Method –** Need to restructure the method section – I suggest having the ‘objective’ section before the method so that you can put the general and specific objectives. The objectives are fragmented and there are lots of redundancies.

**Although we have kept the overall structure similar, the objectives and outcomes are now at the start of each tier section, and redundant text has been cleared to reduce any repetition. We hope this is clearer for the reader.**

**L130 – 138**, Move the paragraph about the “overview of VERN” into the introduction part. Probably at the end of the introduction and before method.

**This has been moved**

**L139-141** – Move to the objective section or just put it at the end of the introduction.

**The study overview, with a brief description of the aims has been moved to the end of the introduction.**

Strat method with L141 “ This study has 3 tiers ….”

**Changed**

**L152** – Once you put the general and specific objectives for each tier above, you may not have to repeat the objectives for each tier. If you prefer to put under each tier, just avoid repetitions.

**This has been edited to reflect the reviewer’s comments**

**L152** - I think if there is no secondary objectives or secondary outcomes, no need to level as ”Primary”. Apply this for the following tier as well.

**The objectives have been clarified and broken down into clearer primary and secondary objectives**

**L166 -179** – Too many outcomes and some of these are activities that may define the listed outcomes. For example, conducting multi-disciplinary team meetings is an activity.

**The primary outcome has been clarified to include ‘activities’. The team feel that for those departments who use the MDT, it is a separate activity to the other processes included in the primary outcome list.**

**L185-189** – See comments above about objectives for each tier.

**Addressed under comments above**

**L193-197,** Patient enrolment

- How about the exclusion criteria? Do you have any?

**Details of inclusion and exclusion criteria have been added to the tier 2 and 3 sections to make it clearer for the reader.**

- Will you include unknown status for COVID-19? Refusals for testing?

**Yes. COVID-19 status is not part of the inclusion or exclusion criteria. We are not documenting reasons for lack of testing formally.**

- You are to include prospective patients.

**Yes, although to ensure comprehensive data capture, patients may also be identified retrospectively.**

- How do you minimize providers' bias?

**The role of principle investigator will be to ensure the protocol is followed, and that sequential patients are included in the study, in order to prevent this bias. This will be according to priciples documented in the declaration of Helsinki.**

- Will you let the providers/Vascular specialists know about the study?

**Yes. Details of this were included in the materials and methods section, but have been moved up to the study overview section for clarity.**

**L216-224** – You may need to have an “Operational definition” section to define the outcome variables. For example,

- How do you measure morbidity? Morbidity related to Vascular disease, related to intervention, COVID-19, or co-morbidities?

**Both will be measured. A full list of morbidity and mortality measures have been included in the supplementary material for reference.**

- How many days of sickness is considered as morbidity? Measure subjectively or objectively?

**Number of days for a sickness won’t be included, just the record of a morbidity or complication.**

- The same thing does for Re-admission.

**As above, number of days is not a factor (we are not planning a cost analysis), only the fact that a re-admission occurred.**

- Within how many days after discharge is considered as re-admission?

There is no time limit applied to re-admission, it will reflect its link to the index procedure of presenting complaint.

- With the same case or re-admission for any case?

**The following statement has been added to the sections for tiers 2 and 3:**

**“Any type of re-admission will be included during the follow-up in the clinical team consider it is linked to index [procedure (tier 2) OR referral (tier 3)]. Morbidity will be defined by condition specific complications and generic surgical and medical complications in the clinical report forms. A full list of outcomes to be measured at six months and one year have been included in the supplementary material.**

**L234** – You will invite vascular specialists only to complete the survey. However, the burden of COVID-19 also affects other professionals like nurses, anesthesiologists, and surgical technologists. Why do you propose to include only vascular specialists in this study?

**We assume this is in reference to Tier 1 – From our collaborative work, and communications with colleagues in other specialties we are aware of a multitude of other pieces of work designed to address the impact of the pandemic on our nursing, anaesthetic and allied medical colleagues. We do not want to repeat surveys already addressed by other specialties. Our focus is on the vascular community as we know it well, and hope to produce work that will help us all directly.**

**Sampling and sample size determination** - Although it is stated under limitation, it is better to explain how to include participants for both patients and vascular specialists. You can also put a sample size but justify it.

- Do you have any inclusion criteria for the Vascular Specialists?

**The protocol has been updated with inclusion criteria for tiers 2 and 3.**

- How do you control/consider cofactors?

**This is a non-interventional study, and one of its’ nature has not been performed before, therefore we have no sample size. We will be describing and presenting results, and do not plan to perform any in depth control of cofactors at this stage. Once 50 patients have been entered we will review the data and assess if more details analysis is appropriate.**

- Do you think all the situations, except related to COVID-19, are comparable pre- and during the pandemics?

**This has depended on the results of tier 1. Thus far, our results have shown that there have been major changes to how vascular surgery is practiced globally. This applies to patients with and without COVID-19. Therefore we believe it is reasonable to compare all factors of care occurring during the pandemic with those aspects during equivalent times in previous times.**

- Please put some assumptions that will justify your comparative analysis before and during the COVID-19 pandemics.

**We have made no assumptions in this protocol, as this is an unprecedented situation. We will review our data after the first 50 patients to establish the assumptions we will make during analysis. We will also review data from other studies that are published in other specialties to establish relevant variables to include in outcome analysis.**

**References**

- Some references missed the publication date or accessed date. Reference 1&12.

**This has been updated**

- I can't see the in-text citation for references 12&13. I am not sure but just check all the in-text citations appear in the reference lists and vice-versa.

**This has been checked and updated**
